# A Case of Cystic Goitre1Read at the 37th annual meeting of the Medical Association of Central New York, held at Rochester, N. Y., September 20, 1904.

**Published:** 1905-04

**Authors:** Nathan Jacobson

**Affiliations:** Syracuse, N. Y. Professor of Clinical Surgery in the College of Medicine, Syracuse University; Surgeon to St. Joseph’s Hospital


### BUFFALO MEDICAL JOURNAL.

VOL. XLIV.—LX. APRIL, 1905. NO. 9

### ORIGINAL COMMUNICATIONS.

### A Case of Cystic Goitre.[1]

BY NATHAN JACOBSON, M. D., Syracuse, N. Y.
Professor of Clinical Surgery in the College of Medicine, Syracuse University; Surgeon to St. Joseph's Hospital.

I DESIRE to occupy but a few minutes of your time in the presentation of the history of a case of cystic goitre:

The patient, a boy of 18, who was attending school, was brought to me on the 22d of March, 1904, by his physician, Dr. Kaple, of Elbridge. His father came with him also. The boy had had measles and whooping cough, but otherwise had been free from the diseases of early life nor had he ever been seriously sick. When but eight years of age his neck began to enlarge in the median line and the growth had steadily increased in size since that time. During the past two years the increase had been greater than during the preceding eight; and in fact during the past six months it had been particularly rapid. He suffered no great distress in breathing except when his head was carried well backwards.

Examination showed the presence of a smooth tumor with an irregular surface which was elastic and in which fluctuation could be made out. The mass was placed largely to the right side of the median line. At its most prominent part the circumference of the neck was 17½ inches. With a hypodermic syringe introduced into the tumor some bloody fluid was withdrawn. The diagnosis of cystic goitre was made and radical operation advised.

An interesting point in the history of the case was the fact that the boy's father had a goitre, apparently of the adenomatous type. These were the only two instances in the family.

1. Read at the 37th annual meeting of the Medical Association of Central New York, held at Rochester, N. Y., September 20, 1904.

The patient did not consent to operation until April 26, 1904. On the preceding day he was admitted into St. Joseph's Hospital, Syracuse. There was an apparent increase in the tumor during the five weeks which had intervened. I was assisted in the operation by Dr. Coon and the hospital internes. A curvilinear incision was made beginning at the angle of the jaw on the right side and ending over the sternal notch. This was deepened through the superficial muscular structures overlaying the tumor. The sternomastoid was pushed back. The outer border of the sternohyoid was cut through. A number of greatly enlarged veins, some of them with a diameter of ¼ of an inch, were doubly ligated and severed between the ligatures. All bleeding was controlled and the cysts were then shelled out separately with the handle of a knife or the finger. Most of them presented no difficulty in their enucleation. The deepest one was firmly attached to the right lobe of the thyroid gland and with it a part of the gland was removed. The right recurrent laryngeal nerve could be readily recognised and injury to it was, therefore, easily avoided. About thirty vessels were ligatured. All of the cysts were removed intact with a single exception and this, though ruptured, was entirely enucleated. Three strips of folded gauze ½ inch in width were packed in the resulting cavity. The wound was closed with interrupted silkwormgut sutures.

Throughout the operation the head was steadied by an assistant so as to secure it in a position in which respiration could be least disturbed. Chloroform was the anesthetic used. The patient bore the operation well.

In the subsequent history there was nothing particularly disturbing. Twenty-four hours after operation the pulse, which had been below 100, rose to 120 and on the second day to 150. During this period there was an increase in the temperature to 102°. May 1, five days after operation, the temperature dropped to 99° and the pulse ranged from 90 to 100. He had some difficulty in swallowing and was unable to move his head from side to side. By the fifth day after the operation, this also had cleared up and from this time on he had but little discomfort. There was from the very start a large amount of serous discharge which steadily decreased in quantity. On May 11 he was able to be up and on the 14th returned to his home; having been in the hospital nineteen days. The boy has remained in perfect health since his return home. I present to you the cysts which were removed. You will note that there are eight in number. They vary in circumference from 2 inches to 7½ inches and in diameter from ¾ of an inch to 2½ inches.

While it has been my privilege to operate upon a number of cases of cystic goitre, the one presented for your consideration contained the largest number of cysts I have ever removed from a neck.

It seems to me that the case has a number of interesting features associated with it. Diseases of the thyroid gland much more frequently affect women than men. To encounter, therefore, a family in which only the males are affected is certainly unusual. What to me seems quite as rare is the appearance of this disease so early in life, beginning as it did with our patient when he was but 8 years of age. There is no doubt whatsoever that these are true cysts of the thyroid gland. In my experience also, single cysts of the thyroid are much more frequent than multiple ones. In our patient the mass, because of its size, did not move upwards and downwards with the act of deglutition. With the aid of a hypodermic syringe there was no difficulty in establishing the diagnosis.

There may be some difference as to the best method of treating a single cyst, especially where the walls thereof are calcareous and fixed and where the enucleation of it would be very difficult and perhaps attended with great danger. Such a case might possibly do better with incision and drainage. But where enucleation is possible and especially where one has to deal with multiple tumors, nothing but complete enucleation is to be considered. With care as to hemorrhage and avoiding unnecessary traction so as not to produce kinking of the trachea, these operations are attended with an exceedingly low mortality. Our patient despite the size of the tumor presented no special difficulty in the way of anesthesia. While there is a growing preference for local anesthesia in these cases, I have always obtained good results with chloroform as the anesthetic. The advantage of absolute quiet on the part of the patient, which can only be obtained with general anesthesia, is apparent. It is admitted that cocaine does not absolutely control the pain and it surely does not overcome the anxiety and nervousness of the patient. As to the safety of chloroform in operations for goitre, it is only necessary to remind you that Kocher has used it in 900 cases without a death. The febrile disturbance and tachycardia which were present after operation, are encountered in practically all of the cases of goitre as a postoperative manifestation.

430 S. SALINA STREET.

DISCUSSION.

Dr. J. P. CREVELING, Auburn.—The case presented by Dr. Jacobson is certainly very interesting, and there are more and larger cysts there than I have ever seen taken from a neck, and it is of much interest in that way. I endorse what Dr. Jacobson has said in reference to anesthesia. I very much prefer chloroform to cocaine. Cocaine does not relieve nervous anxiety nor tension, but which are entirely overcome by chloroform, and these are items of consideration when cutting around a neck well supplied with vessels, with every motion of the patient liable to bring the knife in contact with very important structures. I have operated a number of times, removing the entire gland, which is a serious operation and should be done very carefully, plenty of time being taken to ligate all vessels; also, caution should be taken not to get outside of the margin of the gland. It is important, in my view, to be careful about not removing the entire gland, serious effects having been observed when the entire gland has been removed. A case now occurs to me on which I operated some six or eight months ago, one half of the gland being removed. Since that time the other half has contracted or lessened very much, until it gives the individual now no especial discomfort whatever.

Dr. D. M. Totman, Syracuse.—I had in my earlier practice a case of cystic goitre, in which the operation consisted simply of making an incision into the goitre. It was a single sac occupying the front of the neck quite fully. The patient was a woman about 40 years of age who, when a girl about 12 or 14 years of age, had been injured in running across a yard by striking her neck with a clothesline. This cyst in the neck had been aspirated two or three times by a homeopathic physician in Syracuse, but it repeatedly filled up with blood. It contained bloody fluid when I first saw it and the woman was in desperate straits for her life. The incision was made in the presence of Dr. Jacobson and Dr. Elsner; immediately there was a gush of bloody fluid from the sac, which immediately collapsed. Dr. Elsner examined at once, and exclaimed, "There are other tumors." This quite surprised me. The quantity of blood which gushed out was very large, but the tumors instantly disappeared. Evidently this cyst had no lining membrane, except dilated bloodvessels; these immediately filled with blood, then burst, causing enormous hemorrhage. I emptied the tumor of the blood, and filled the cavity with peroxide of iron pledgets, put on a compress as tight as possible and permit her to breathe, and the case went on to perfect recovery.

Dr. Young.—Will Dr. Jacobson kindly state whether, as a rule, these cysts are tubercular or not?

Dr. Jacobson, closing the discussion.—With reference to the remarks that have been made in the discussion of this paper, first, let me speak as to the class of cases to which Dr. Totman refers. In his case, which I recall very vividly, the history was that of an injury to the thyroid, and the operation, opening up into a sac which gave vent to a large quantity of bloody fluid and blood, showed the case to be of the other type of cysts—namely, false cysts of the thyroid. In others there is a clean cyst wall, with the dense capsular formation, the contents of which can be shelled out. These are the true cysts. On the other hand, there is a class of cases in which there has been a hemorrhage into the substance of the thyroid gland, there being no true cyst wall, but simply a large space distended because of hemorrhage, these being the false cysts of the thyroid gland. Those are the two types we encounter.

I know nothing of the tubercular form of cysts of the thyroid. I doubt if such a condition as that exists in the thyroid. In other tumors of the thyroid gland—and now since the thyroid has been operated upon so much we know more about it,—we know that there are distinct conditions which differ absolutely the one from the other, and these types which we encounter most frequently, and the one which is quite as amenable to surgical treatment as any, is the form in which we have a tumor built up in the thyroid gland, which has a distinct wall. Therefore, the surgeon should be careful to ligate the vessels in advance in this form. The large veins and the inferior thyroid artery, in the other form, can be shelled out without much hemorrhage.

I recall in the winter of 1877-8 when I first witnessed operations on the thyroid gland, there was no operation which was as bloody as the operation done at that time, but, with the advance in surgical methods and with the appreciation of what we are dealing with in these cases of so-called goitre, we recognise a vast difference in our pathology and the surgical treatment is changed accordingly. There is no question at all that so-called goitre is a disease which assumes many variations, most of which are amenable to surgical treatment, and the thousands of cases which have been operated upon in Switzerland by Couvier, and others who have had much experience there, show the mortality is exceedingly small. Couvier has operated, perhaps two thousand times, with a mortality of a very few per cent., thus demonstrating these cases to be amenable to surgical treatment with small danger. Again, there is another class of goitre cases in which there is simply the enlargement of the structures. In these reduction in the size of the tumor can be secured by means of medicinal and other methods. But the cases of true tumor of the thyroid, which are cysts, demand surgical operation, and those cases can be operated upon safely and easily. The other class of cases, parenchymatous goitre for which some form of iodine can be administered or the thyroid extract, are amenable to medicinal treatment; but, as I have remarked before, when the true cysts or tumors are encountered you must operate. As to results in the cases in which excision and drainage is employed, you have to deal with another feature—namely, repair by granulation. In such cases, where you pack with gauze or cotton saturated with the iron preparations, you have to deal with a condition that leads to suppuration; a wound which heals but slowly. In clean operations prompt repair takes place.

